# Co-targeting EGFR and IKKβ/NF-κB signalling pathways in head and neck squamous cell carcinoma: a potential novel therapy for head and neck squamous cell cancer

**DOI:** 10.1038/s41416-018-0351-z

**Published:** 2018-12-26

**Authors:** Zhipeng Li, Jipei Liao, Zejia Yang, Eun Yong Choi, Rena G. Lapidus, Xuefeng Liu, Kevin J. Cullen, Hancai Dan

**Affiliations:** 1 Marlene and Stewart Greenebaum Comprehensive Cancer Center, University of Maryland School of Medicine, Baltimore, MD USA; 2 Department of Pathology, University of Maryland School of Medicine, Baltimore, MD USA; 3 Department of Pathology, Georgetown University Medical Center, Washington, DC USA

**Keywords:** Cancer, Translational research, Growth factor signalling

## Abstract

**Background:**

Epidermal growth factor receptor (EGFR) plays an important role in head and neck squamous cell carcinoma (HNSCC) proliferation and therapy resistance, but the efficacy of targeting of EGFR for therapy has been limited. Here, we explore the molecular link between EGFR and inhibitor of κB kinase β/nuclear factor-κB (IKKβ/NF-κB) signalling pathways in the regulation of HNSCC EGFR inhibitor resistance.

**Methods:**

We performed in vitro experiments in eight human HNSCC cell lines and a patient-derived HNSCC cell line as well as in vivo xenografts in a human HNSCC cell line.

**Results:**

We found that treatment of all HNSCC cells with Gefitinib and Erlotinib, two Food Drug Administration-approved EGFR inhibitors, blocked the activity of Akt/mammalian target of the rapamycin (mTOR) and extracellular signal-regulated kinase, two crucial downstream effectors of EGFR, but up-regulated IKKβ/NF-κB signalling. In addition, induction of IKKβ/NF-κB by EGFR inhibitors required HER2 and HER3 expression. In keeping with these, IKKβ inhibitor CmpdA synergistically enhanced the efficacy of EGFR inhibitors to further inhibit in vitro HNSCC cell growth. Importantly, we demonstrated that the combination of Gefitinib with CmpdA inhibited xenograft tumour formation.

**Conclusion:**

Our data demonstrated that co-targeting EGFR and IKKβ with Gefitinib and IKKβ inhibitors could provide a potential novel therapy for head and neck squamous cell cancer.

## Introduction

Head and neck cancers rank as the sixth most common cause of human cancer deaths in the world, which results in roughly 300,000 deaths per year.^1,2^ Most cases are head and neck squamous cell carcinomas (HNSCCs) that mainly encompass cancer from the oral cavity, pharynx (nasopharynx, oropharynx and hypopharynx) and larynx.^3,4^ Surgery or radiation therapy achieves excellent outcomes for early-stage HNSCC. However, treatment success is more limited in patients with late-stage HNSCC, where the cancer progresses with significant loco-regional invasion and lymph node metastasis.^4–6^ Cisplatin-based chemotherapy is currently the most common treatment protocol for HNSCC and is most often combined with radiation therapy. Moreover, it is the only treatment option for individuals with recurrent and metastatic HNSCCs. Unfortunately, patients with local relapse in the radiation field or with distant metastasis will rapidly develop resistance to this treatment and generally die within one year. Therefore, there is an urgent need to explore novel therapies that will improve patient survival rates.^4–7^

Targeted therapy has been an exciting advance in the treatment of several cancer types, including those of head and neck. HNSCC proliferation and therapy resistance are regulated, in part, through signalling of the epidermal growth factor receptor (EGFR). Therefore, use of the EGFR antibody, Cetuximab or small-molecule EGFR tyrosine kinase inhibitors, Gefitinib and Erlotinib, to target the receptor has been well studied.^8–10^ Although Cetuximab is an approved HNSCC therapy, the overall impact on survival is modest.^11,12^ Moreover, Cetuximab is currently extremely expensive, which creates a major hurdle towards widespread treatment. Gefitinib and Erlotinib have been used in lung cancer treatments,^13,14^ and clinical trials in HNSCC have demonstrated that supplementation of cisplatin-based chemotherapy with Gefitinib or Erlotinib will improve the quality of life, but not patient survival rates.^15,16^ Many studies have indicated that induction of some compensatory survival signalling pathways can limit EGFR inhibitor therapy success.^17–20^

EGFR regulates HNSCC survival, proliferation, migration, invasion, metastasis and chemotherapy resistance through several crucial downstream targets. These include the phosphatidylinositol-3-kinase (PI3K)-Akt-mammalian target of the rapamycin (mTOR) pathway, the extracellular signal-regulated kinase 1/2 (ERK1/2) pathway and the Janus kinase/signal transducers and activators of transcription (STAT) pathway.^4,8–10^ These pathways regulate tumourigenesis through translational or transcriptional regulation of their downstream target(s). Important effectors of those pathways are the members of the transcription factor nuclear factor-κB (NF-κB) family: p65 (RelA), RelB, c-Rel, p50/p105 (nuclear factor NF-κB1) and p52/p100 (NF-κB2). These, in turn, are regulated by canonical and non-canonical NF-κB pathways.^18,19,21–24^ In the canonical pathway, inhibition by IκBα results in an inactive p65 (RelA)-p50 heterodimer. When a key upstream kinase of NF-κB, comprised of two catalytic subunits, inhibitor of κB kinase α and β (IKKα and IKKβ) and a regulatory subunit, IKKγ/NEMO, is activated, it will phosphorylate IκBα. This leads to IκBα degradation and NF-κB activation. The activated NF-κB then regulates downstream cellular processes to promote cancer cell survival, proliferation and resistance to both targeted therapy and chemotherapy.^18,23,24^

We previously reported that EGFR activated the IKK/NF-κB pathway through mTORC1 downstream of PI3K/Akt in both cisplatin-sensitive and cisplatin-resistant HNSCC cells.^25^ Here, we examine the role of IKK/NF-κB in the regulation of HNSCC cell sensitivity to EGFR inhibitors, including Gefitinib and Erlotinib, in multiple HNSCC cell lines in vitro and in vivo. We found that treatment of HNSCC cells with Gefitinib and Erlotinib blocked the activity of downstream effectors Akt/mTOR and ERK, but up-regulated IKKβ/NF-κB signalling. Furthermore, the IKK inhibitor CmpdA enhanced the efficacy of EGFR inhibitors in HNSCC cell growth inhibition in vitro and in vivo. Our findings indicate that IKKβ plays an essential role in the regulation of EGFR inhibitor resistance, and, therefore, co-targeting of EGFR and IKKβ may be an effective treatment strategy for refractory HNSCC.

## Materials and methods

### Cell lines

HNSCC cell lines Cal27, FaDu and SCC25 were purchased from ATCC. UMSCC1, UMSCC6, UMSCC9 and UMSCC11A were the generous gift of Dr. Thomas E. Carey (University of Michigan, Ann Arbor, MI, USA). The Cal33 cell line was from Dr. Jennifer R. Grandis (UCSF School of Medicine). All cells were maintained in Dulbecco’s modified Eagle’s medium (DMEM) supplemented with 10% foetal bovine serum, 2 mM glutamine and 100 U/mL penicillin and streptomycin (Gibco). A patient-derived HNSCC cell line was established in Dr. Xuefeng Liu’s laboratory at Georgetown University Medical Center, Washington, DC, USA (Protocol: A328201, Georgetown University Medical Center). The patient was a 60-year-old white female with stage T3N0M0 tongue squamous cell carcinoma. It is important to note that the patient had not received any chemotherapy or radiation therapy before the tissue was obtained for cell isolation.

### Reagents and antibodies

Protease and phosphatase inhibitor cocktails were purchased from Roche and CHAPS was obtained from Pierce. Gefitinib and Erlotinib were from Selleckchem. The IKKβ inhibitor, CmpdA, was a gift from Dr. Albert Baldwin at the University of North Carolina (Chapel Hill, NC, USA).

Antibodies against phospho-EGFR-Y1068 (CST-3733), phospho-HER2-Y1248 (CST-2247), HER2 (CST-4290), phospho-HER3-Y1289 (CST-2842), HER3 (CST-12708), phospho-p65-S536 (CST-3033), p65 (CST-6956), phospho-IKKα S176/β S177 (CST-2697 and CST-2078), IKKα (CST-2682), IKKβ (CST-8943), phospho-Akt-S473 (CST-4508), Akt (CST-2938), phospho-S6K-T389 (CST-9205), S6K (CST-9202), phospho-ERK-T202/Y204 (CST-4370), ERK (CST-4348), cleaved caspase-3 (CST-9664) and GAPDH (CST-5174) were purchased from Cell Signalling. Anti-EGFR (SC-03) came from Santa Cruz Biotechnology, along with horse radish peroxidase-labelled anti-mouse and anti-rabbit secondary antibodies.

### Cell lysis and western blot analysis

Cells were lysed and immunobotted as described previously.^25^ As needed, densitometric analyses of Western blot bands were performed using the ImageJ software.

### Co-Immunoprecipitation experiments

For co-immunoprecipitation (co-IP) experiments, 4 μg of each antibody was added to cell lysates and incubated with rotation at 4 °C for 6–16 h. Then, 25 μL of protein G-agarose were added, followed by an additional 1-h incubation. Immunoprecipitates were washed three times with lysis buffer and boiled in 4× SDS sample buffer for 5 min prior to electrophoresis and immunoblotting.

### NF-κB reporter assay

Cells were seeded in six-well plates. The next day, cells were transfected with 200 ng of 3× κB luciferase reporter and 50 ng of pRL-SV40 (*Renilla* reporter control) DNA. After a 24-ho incubation, cells were treated with Gefitinib (5μΜ) for an additional 24 h. Cells were harvested, and luciferase assays were performed using the Dual Luciferase Assay System (Promega) as per the manufacturer’s instructions. The experiments were performed in triplicate.

### siRNA transfection

Small interfering RNA (siRNA) HER2 and HER3 reagents were purchased from Santa Cruz Biotechnology. The non-targeting siRNA was from Dharmacon. Cells were transfected with indicated siRNA or nonspecific control pool using DharmaFECT 1 reagent (Dharmacon) according to the manufacturer’s instructions and as described previously.^25^ Cells were treated with the indicated inhibitors 24 h after siRNA transfection and harvested 48–72 h after siRNA transfection.

### Cell proliferation assays

Cells were plated in 96-well plates in triplicate at 3 × 10^3^ cells per well and cultured in the presence or absence of Gefitinib or the IKKβ inhibitor with indicated concentrations and time courses. At the end of each time point, 3-(4,5-dimethylthiazol-2-yl)-5-(3-carboxymethoxyphenyl)-2-(4-sulphophenyl)-2*H*-tetrazolium (MTS) compound (Promega, Cat#: G3580) was added and incubated at 37 °C for 1 h prior to colorimetric readouts at 490 nm on a Versamax Microplate Reader (Molecular Devices). The combination index values were determined according to the Chou–Talalay method^26^ using the CalcuSyn software.

### Caspase activity

Caspase activity was measured as described previously.^25^ Briefly, cells were plated in triplicate at 2 × 10^3^ cells per well in 96-well plates (Becton Dickinson) for 24 h and then treated with IKK inhibitor and/or EGFR inhibitor for an additional 48 h. Caspase-3/7 activity was measured using the Caspase-Glo-3/7 assay (Promega) according to the manufacturer’s instructions.

### Gene expression profile

RNA from Cal27 and Cal27GP cells was isolated according to miRNeasy Mini Kit (Qiagen) and gene expression was analysed using GeneChip^@^ HuGene 2.0 ST Array. Dr. Yuji Zhang (Department of Epidemiology and Public Health, University of Maryland Marlene and Stewart Greenebaum Cancer Center) performed the statistical analysis.

### Colony formation assay

Cells were plated in 12-well plates. The next day, 1 × 10^3^ cells per well were seeded in a 12-well plate, pre-treated with dimethyl sulfoxide (DMSO) or CmpdA for 2 h, followed by Gefitinib treatment for 3 days. Cells were then incubated in normal media for an additional 24 h and allowed to form colonies for 10 days. The plates were then gently washed with phosphate-buffered saline and colonies were stained with crystal violet. Each experiment was performed in triplicate.

### Tumour xenograft formation in mice

Nu/nu mice (Envigo, Frederick MD) were injected subcutaneously on the right flank with 0.5 × 10^6^ FaDu cells in the presence of 33% Matrigel^TM^ (Fisher Scientific). When tumours reached approximately 100 mm^3^, half the mice were pre-treated with 10 mg/kg CmpdA (every other day). When tumours measured approximately 200 mm^3^, mice were divided into four groups of seven mice each. CmpdA was dosed 3 days per week by intraperitoneal (IP) injection and Gefitinib was dosed orally daily until termination of the experiment. Tumour volume was measured twice per week using electronic calipers and animals were weighed 5 days per week. Tumour volume was calculated as (*l* ×*w*^2^)/2, where *w* is the smaller dimension. Mice were euthanised on day 14 of the study, and the tumours were excised, weighed, fixed and frozen. Studies were performed with Institutional Animal Care and Use Committee approval (protocol 1016012).

### Statistics

Data from in vitro experiments were expressed as mean ± SE using a minimum of three independent experiments. Comparisons between groups were carried out by two-way analysis of variance or Student’s *t* -test. For mouse studies, the two-tailed *t* -test was used to compare tumour volumes and weights between control and treatment groups. *P* values <0.05 were considered significant.

## Results

### Inhibition of IKK/NF-κB signalling improves the efficacy of EGFR inhibitors in HNSCC cells in vitro

**Fig. 1:**
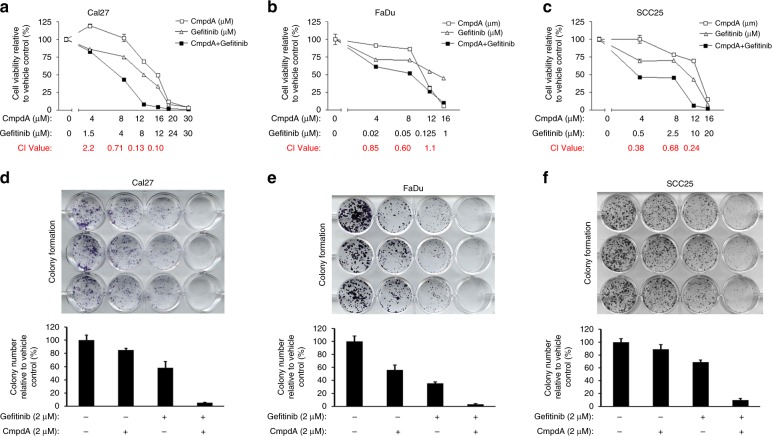
Inhibition of cell proliferation by co-targeting EGFR and IKK in HNSCC cells. **a**–**c** Gefitinib and IKKβ inhibitor CmpdA synergistically inhibit cell proliferation. Cal27 (**a**), FaDu, (**b**) and SCC25 (**c**) cells were treated with DMSO, Gefitinib, CmpdA or a combination for 72 h and cell proliferation was determined by the MTS assay. The experiments were performed in triplicate, and the results are representative of three independent experiments. The combination index values (CI values) were determined using the CalcuSyn software. **d**–**f** Synergistic inhibition of colony formation by Gefitinib and CmpdA combination. Cal27 (**d**), FaDu, (**e**) and SCC25 (**f**) cells were treated with DMSO, Gefitinib, CmpdA or a combination for 24 h and colony formation was observed 10 days after treatment. Each experiment was repeated three times

We used a well-characterised selective IKKβ inhibitor CmpdA (also named Bay 65-1942) that significantly blocked IKK phosphorylation of NF-κB in multiple cancer cells^27^ to determine whether blockage of the IKK/NF-κB pathway activity sensitised HNSCC cells to EGFR inhibitor treatment. Cal27 cells were treated with DMSO control as well as increasing doses of either Gefitinib or CmpdA, or a combination for 72 h. Cell proliferation was measured by MTS assay and cell viability was normalised to the DMSO control. As shown in Fig. 1a, treatment with Gefitinib or CmpdA led to dose-dependent inhibition of cell proliferation; however, their combination increased inhibition of cell proliferation compared with single treatments (Fig. 1a). Similarly, Gefitinib or CmpdA also inhibited FaDu and SCC25 in a dose-dependent manner, while the combination enhanced these effects (Fig. 1b, c). In order to further determine whether a combination of Gefitinib and CmpdA caused synergistic inhibition of cell proliferation, we employed the CalcuSyn software to analyse combination index (CI) value according to the Chou–Talalay method.^26^ CI values from a majority of the combined inhibitor doses were <1 in all cell lines (Fig. 1a–c), which indicated a strong synergism between Gefitinib and CmpdA. We next performed colony formation assays under different conditions. As shown in Fig. 1, a combination of CmpdA and Gefitinib significantly reduced the colony number compared to either agent alone in Cal27 (Fig. 1d), FaDu (Fig. 1e) and SCC25 (Fig. 1f) cells. We also found that the combination of CmpdA and Erlotinib visually reduced colony formation compared to CmpdA or Erlotinib alone in Cal27 (Supplementary Figure 1A) and FaDu (Supplementary Figure 1B) cells. Taken together, these data indicate that CmpdA synergistically sensitised HNSCC cells to Gefitinib and Erlotinib treatment.

### EGFR/IKKβ co-targeting through a combination of Gefitinib and CmpdA suppressed xenograft tumour formation in mice

**Fig. 2:**
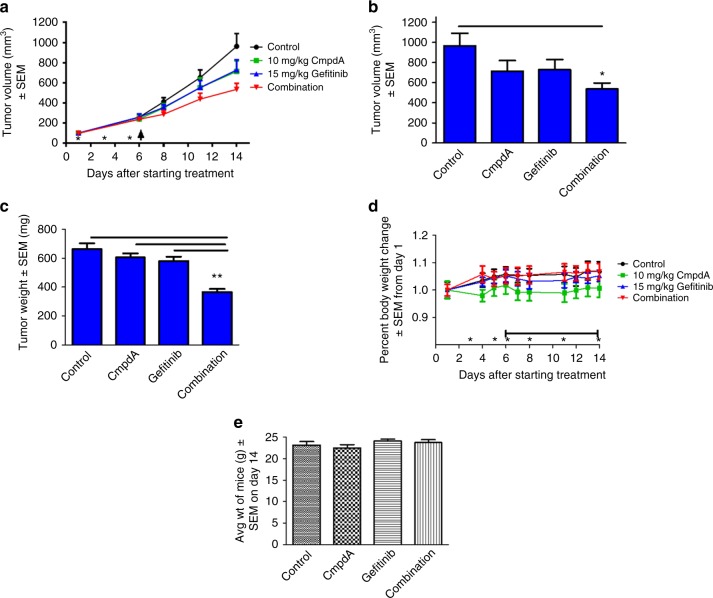
Synergistic inhibition of FaDu cell xenograft tumour formation by a Gefitinib/CmpdA combination. **a** Inhibition of xenograft tumour formation was measured by tumour volume. Mice inoculated with FaDu cells were randomised into four groups for 2-week treatments: (i) DMSO control; (ii) Gefitinib (15 mg/kg, PO); (iii) CmpdA (10 mg/kg, IP) and (iv) Gefitinib plus CmpdA. Tumour growth curves based on tumour volumes were measured twice weekly (*pre-treatment with CmpA; ↑onset of Gefitinib treatment). **b** Final tumour volumes were compared at the time of sacrifice. **c** Final tumour weights were measured and compared at the time of sacrifice. **d** Mouse body weights were monitored 5 days per week. **e** Final body weights were measured at the time of sacrifice (right). *p<0.02 vs. control; **p<0.03 vs. control, CmpdA, Gefitinib vs. combination

We evaluated the in vivo antitumour activity of Gefitinib and CmpdA in combination using mouse xenografts. FaDu cells were inoculated into mice. When tumours reached approximately 200 mm^3^, mice were randomised to one of four treatment groups: vehicle control (*n* = 7), 15 mg/kg, Gefitinib by per os (PO) (*n* = 7), 15 mg/kg CmpdA by IP (*n* = 7) or a combination of Gefitinib and CmpdA (*n* = 7). We originally intended to treat mice for 4 weeks, but we had to terminate the experiment on day 14 due to tumour necrosis in the most control mice and several single treatment mice. The average means of tumour volumes of Gefitinib or CmpdA treatment groups were lower than that of the control group, but there was no significant difference in tumour volumes between single treatment (either Gefitinib or CmpdA treatment) vs. control or combination groups due to the tumour necrosis (Fig. 2a, b). However, tumour volumes in the combination treatment group were significantly lower than tumours in the control group by the end of the study (Fig. 2a, b). Similarly, when tumours were excised and weighed at the end of the study, there was no significant difference in tumour weights (mg) between single treatment (either Gefitinib or CmpdA treatment) vs. control groups (Fig. 2c). Most likely, this can be explained by the tumour necrosis observed in the control and single treatment groups (Fig. 2c). Importantly, tumour weight in the combination treatment group was significantly lower compared with the mice treated with vehicle, Gefitinb or CmpdA alone (Fig. 2c). In summary, as a single agent, both Gefitinib and CmpdA had modest inhibitory effects on tumour growth in vivo, and a combination of the two drugs led to increased inhibitory effects on tumour growth (Fig. 2a–c). This suggests that dual inhibition of EGFR and IKK through Gefitinib and CmpdA synergy more effectively suppressed tumour growth in mice. Importantly, all single and combined doses of CmpdA and Gefitinib were tolerable because there was no significant weight loss observed during the study (Fig. 2d, e).

### EGFR inhibitors up-regulated IKK/NF-κB signalling through HER2 and HER3 in HNSCC cells

**Fig. 3:**
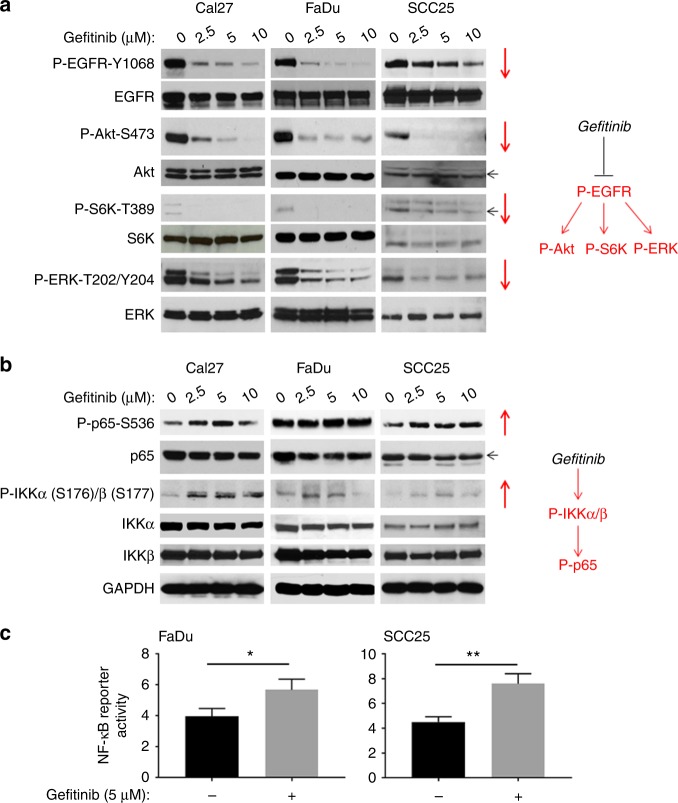
Gefitinib inhibits EGFR, Akt, mTOR and ERK pathways, but induces IKKβ/NF-κB signalling in HNSCC. Gefitinib induction of IKKβ/NF-κB signalling involves HER2 and HER3. **a**, Left panel: inhibition of EGFR, Akt, mTOR and ERK phosphorylation by Gefitinib. HNSCC cell lines Cal27, FaDu and SCC25, were treated with vehicle control (DMSO) or Gefitinib (2, 5 or 10 μmol/L) for 24 h. Phosphorylation status and total protein levels of EGFR, Akt, S6K, ERK, IKKα/β and p65 were analysed by Western blot. The results are representative of three independent experiments. Right panel: a diagram that shows Gefitinib inhibition of EGFR, Akt, mTOR and ERK. **b**, Left panel: induction of phosphorylation of IKK and NF-κB (p65) by Gefitinib. Cell lysates from Fig. 3a were used for Western blot analysis to test phosphorylation status and total protein levels of IKK and p65. Right panel: a diagram that depicts Gefitinib up-regulation of IKKβ/NF-κB signalling. **c** Gefitinib induced NF-κB reporter activity in FaDu and SCC25 cells. Cells were transfected with 200 ng of 3× κB luciferase reporter and 50 ng of pRL-SV40 (*Renilla* reporter control) DNA for 24 h, followed by Gefitinib treatment (5 μΜ) for an additional 24 h. NF-κB reporter activity was measured. The experiments were performed in triplicate

We evaluated the effects of Gefitinib treatment on the phosphorylation and total protein expression of EGFR, Akt, S6K (a downstream target of mTOR) and ERK in multiple HNSCC cell lines. Cal27 cells were first treated with the indicated doses for 24 h. As shown in Fig. 3a, the phosphorylation of EGFR decreased in a dose-dependent manner, whereas the total EGFR level remained unchanged (Fig. 3a, left panel). Consistent with the above results, the phosphorylation of EGFR downstream targets Akt, S6K and ERK was also inhibited. Likewise, the total levels of these proteins remained the same (Fig. 3a, left panel). We also examined the effect of Gefitinib on the signalling pathways of two other HNSCC cell lines, FaDu and SCC25. As demonstrated in Cal27 cells, Gefitinib inhibited phosphorylation of EGFR, Akt, S6K and ERK in both cell lines (Fig. 3a, middle and right panels). Taken together, we concluded that Gefitinib treatment led to EGFR inhibition and subsequent inhibition of the Akt/mTOR and ERK pathways in Cal27, FaDu and SCC25 cells (Fig. 3a).

Next, we examined the effect of Gefitinib on the NF-κB signalling pathway using the cell lysates discussed above. Interestingly, we found that Gefitinib treatment elevated NF-κB (p65) phosphorylation (P-p65-Serine 536). Furthermore, we examined the activity of IKK, the major kinase responsible for p65 phosphorylation at Serine 536. The results showed that, similar to NF-κB, phosphorylation of IKKα/β was induced without any change to total protein levels, which indicated that IKK/NF-κB signalling was up-regulated by Gefitinib treatment in Cal27 cells (Fig. 3b, left panel). Similar results were also found in FaDu and SCC25 cells (Fig. 3b, middle and right panels). These results demonstrated that Gefitinib treatment induced IKK and NF-κB (p65) signalling pathways in Cal27, FaDu and SCC25 cells (Fig. 3b). We also treated Cal27 cells with similar doses of Gefitinib for 4 or 48 h and found that treatment with Gefitinib for 4 h could also inhibit EGFR, Akt, S6K and ERK phosphorylation, but, conversely, induced p65 phosphorylation (data not shown).

Analysis in five other HNSCC cell lines (Cal33, UMSCC1, UMSCC6, UMSCC11A and UMSCC38) produced similar results (Supplementary Figures 2A and 2B). Taken together, Gefitinib inhibition of EGFR led to subsequent inhibition of Akt, mTOR and ERK pathways, as well as abnormal up-regulation of IKK/NF-κB signalling in HNSCC cells.

We then evaluated the effects of the EGFR inhibitor Erlotinib on phosphorylation and total protein expression of EGFR, Akt, S6K, ERK, IKK and NF-κB (p65) in Cal27 cell lines. As shown in Supplementary Figure 3, Erlotinib inhibited phosphorylation of EGFR, Akt, S6K and ERK (Supplementary Figure 3A), but enhanced phosphorylation of IKK and NF-κB p65 (Supplementary Figure 3B), while total protein levels remained unchanged (Supplementary Figure 3). Our data demonstrated that EGFR inhibitors induced the IKK/NF-κB pathway in HNSCC cells.

Next, we treated FaDu and SCC25 cells with Gefitinib for 24 h and measured its effect on NF-κB reporter activity. We showed that NF-κB activity was significantly elevated upon Gefitinib treatment (Fig. 3c). These data further confirm that NF-κB signalling was up-regulated by EGFR inhibitor treatment in HNSCC cells.

### HER2 and HER3 levels are elevated upon EGFR inhibitor treatment, and the HER2/HER3 heterodimer is involved in IKK/NF-κB signal up-regulation by EGFR inhibitors in HNSCC cells

**Fig. 4:**
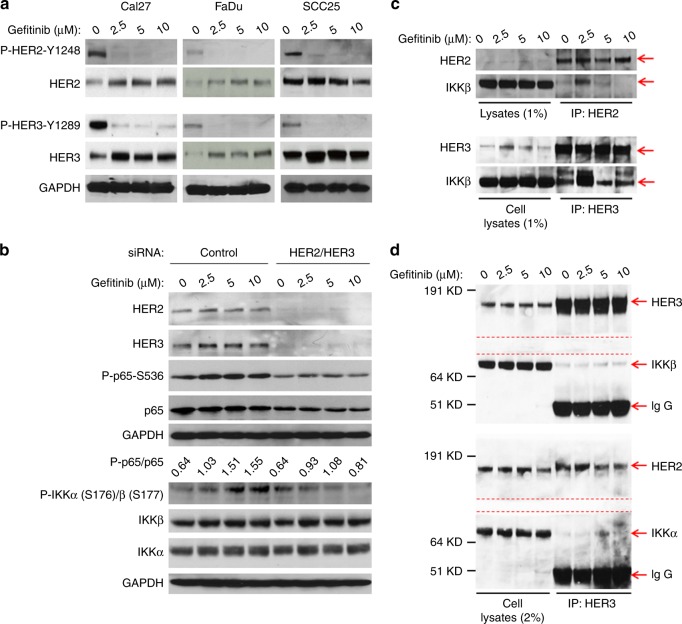
Gefitinib inhibits phosphorylation of HER2 and HER3, but elevates HER2 and HER3 expression; HER2 and HER3 heterodimer associates with IKK and may be involved in Gefitinib-induced IKKβ/NF-κB signalling. **a** Gefitinib inhibits phosphorylation of HER2 and HER3 but induces levels of HER2 and HER3. Phosphorylation and total levels of HER2 and HER3 in the lysates from Fig. 3a were analysed by Western blot. **b** Knockdown of HER2 and HER3 abates Gefitinib-induced IKKβ/NF-κB signalling. Cal27 cells were transfected with non-targeting siRNA or siRNA against HER2 and HER3 for 24 h, and cells were treated with (DMSO) or Gefitinib (2, 5 or 10μmol/L) for 24 h. Phosphorylation status and total protein levels of IKKα/β and p65 were analysed by Western blot. **c** Cal27 cells were treated with increasing doses of Gefitinib for 4 h, lysed and immunoprecipitated with anti-HER2 or anti-HER3 antibodies. Lysates and immunoprecipitates were detected with HER2, HER3 and IKKβ as indicated by Western blot. **d** FaDu cells were treated with increasing doses of Gefitinib for 4 h and lysed. The cell lysates were immunoprecipated with anti-HER3 antibodies. Proteins in lysates and immunoprecitates were resolved by SDS-PAGE, and proteins were transferred to Pure Nitrocellulose Membrane (Bio-Rad), blocked in 5% nonfat milk, and blotted with the indicated antibodies after cutting the membrane as labelled (red lines)

Previous studies reported that EGFR inhibition led to downstream inhibition or induction of other ErbB family members.^17,18,20,28^ We next examined the effects of Gefitinib on HER2 and HER3 phosphorylated and total protein levels. Gefitinib inhibited HER2 and HER3 phosphorylation, but induced total protein levels in Cal27, FaDu and SCC25 cells (Fig. 4a). We then examined whether or not knockdown of HER2 and HER3 by siRNA affected Gefitinib induction of the IKK/NF-κB pathway in Cal27 cells. We found that Gefitinib induced IKK and NF-κB phosphorylation in non-targeting siRNA-transfected cells, but not in cells transfected with siRNA against HER2 and HER3 (Fig. 4b). Importantly, we found that knockdown of HER2 or HER3 alone showed limited effects on IKK and NF-κB phosphorylation compared to HER2/HER3 double knockdown (data not shown). Our data demonstrated that non-phosphorylated HER2 and HER3 were important for Gefitinib induction of the IKK/NF-κB pathway in HNSCC cells.

Next, we determined if there was an interaction between the HER2/HER3 heterodimer and IKKα/β. In Cal27 cells, antibodies against HER2 or HER3 were able to pull down IKKβ in untreated cells, indicating an interaction between the HER2/3 heterodimer and IKK in these cells (Fig. 4c). In addition, we found that 2.5 and 5 μΜ doses of Gefitinib induced this interaction (Fig. 4c). In a parallel co-IP experiment using an IgG control, we were unable to detect IKK (data not shown). Similarly, in FaDu cells, HER3 antibodies were able to pull down HER2, HER3, IKKα and IKKβ; however, cells treated with Gefitinib had more detectable proteins in comparison to untreated cells. These data suggested that there is a constitutive interaction between HER2/3 and IKK, which is enhanced by Gefitinib treatment (Fig. 4d). Our data indicated that Gefitinib induced the interaction between the HER2/HER3 heterodimer and IKKα/β to enhance IKK/NF-κB activity in HNSCC cells.

### Inhibition of EGFR, Akt, mTOR, ERK and NF-κB signalling pathways and induction of apoptosis by co-targeting EGFR and IKKβ

**Fig. 5:**
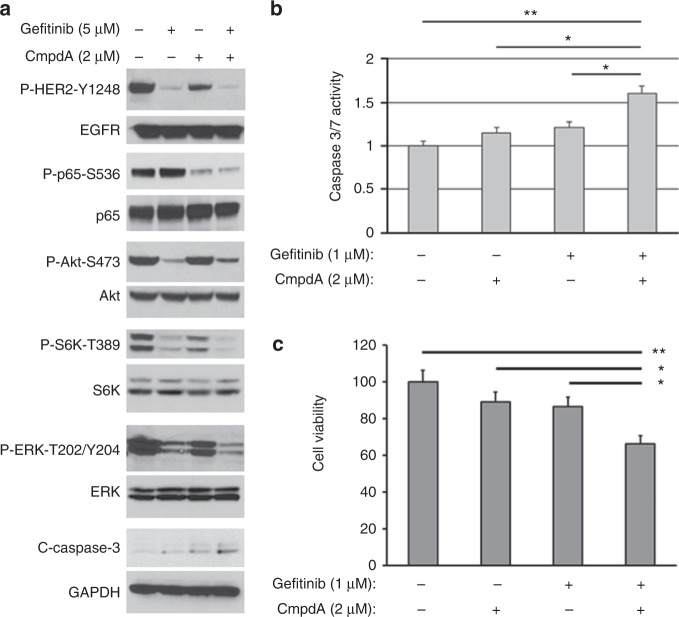
A combination of Gefitinib and IKK inhibitors down-regulate EGFR, Akt, mTOR, ERK and IKKβ/NF-κB signalling pathways and cell proliferation and survival in FaDu cells. **a** Inhibition of signalling pathways with a combination of Gefitinib and IKK inhibitors in FaDu cells. FaDu cells were treated with DMSO, Gefitinib, CmpdA or a combination for 48 h and phosphorylation status and total protein levels of p65, Akt, S6K and ERK, as well as levels of cleaved caspase-3, were analysed by Western blot. **b** A combination of Gefitinib and CmpdA induced more caspase activity compared to either single treatment. Cells were treated with DMSO, Gefitinib, CmpdA or a combination as described above for 48 h and caspase activity was measured (*p < 0.05; **p < 0.01). **c** A combination of Gefitinib and CmpdA significantly inhibited cell proliferation. Cells were seeded in 96-well plates for 24 h and treated with DMSO, Gefitinib, CmpdA or a combination for 48 h as described above. Cell proliferation was determined by the MTS assay (*p < 0.05; **p < 0.01)

We next determined whether CmpdA could block Gefitinib-induced NF-κB activation. FaDu cells were treated with DMSO, Gefitinib, CmpdA or a combination for 24 h, followed by cell lysis and Western blot analysis. Similar to Fig. 3, we found that Gefitinib blocked EGFR, Akt, S6K and ERK phosphorylation, but induced NF-κB (p65) phosphorylation (Fig. 5a). However, addition of CmpdA abolished Gefitinib-induced NF-κB activity, although it slightly attenuated Gefitinib-inhibited Akt phosphorylation (Fig. 5a). Moreover, we detected cleaved caspase-3 in cells treated with either Gefitinb or CmpdA, which suggested that either inhibitor could cause slight cleavage of caspase-3. However, cleaved caspase-3 levels were greatly induced in combined treatment cells (Fig. 5a), which suggested that CmpdA could synergise with Gefitinib to induce FaDu cell apoptosis. Furthermore, we used lower doses of CmpdA and Gefitinib to measure cleaved caspase-3 activity. As shown in Fig. 5b, lower doses of either CmpdA or Gefitinib led to slight increases in caspase activity (*p* > 0.05), while a combination of those doses significantly induced caspase activity (*p* < 0.01; Fig. 5b). In a parallel experiment, cell proliferation was detected by the MTS assay. Low doses of CmpdA (2 μΜ) or Gefitinib (1 μΜ) alone for 48 h did not significantly inhibit cell proliferation (*p* > 0.05), but the combination of Gefitinib and CmpdA dramatically inhibited cell proliferation compared to the control (*p* < 0.01) and CmpdA or Gefitinib alone treatments (*p* < 0.05; Fig. 5c). We repeated the above experiments in both Cal27 (Supplementary Figure 4) and SCC25 (Supplementary Figure 5) cells. CmpdA blocked Gefitinib induction of NF-κB activation and synergised with Gefitinib to induce caspase-3 cleavage (Supplementary Figures 4A and 5A), increase caspase activity (Supplementary Figures 4B and 5B) and inhibit cell proliferation in Cal27 and SCC25 cells (Supplementary Figures 4C and 5C). Taken together, we found that CmpdA enhanced the ability of EGFR inhibitors to induce apoptosis.

### CmpdA improves the efficacy of EGFR inhibitor in patient-derived HNSCC cells in vitro

It is important to define whether CmpdA-induced IKK inhibition could enhance the efficacy of Gefitinib in patient-derived HNSCC cells. To this end, we acquired a patient-derived tongue squamous cell carcinoma cell line recently established by Dr. Xuefeng Liu’s laboratory through the Conditional Reprogram Cell system at Georgetown Medical Center.^29,30^ The cells were incubated in media that contained serum and growth factors for 24 h, followed by treatment with DMSO, Gefitinib, CmpdA or a combination for an additional 24 h. Results from Western blot analyses of cell lysates showed that Gefitinib inhibited EGFR, Akt, S6K and ERK pathways, but increased IKK and NF-κB phosphorylation (Supplementary Figure 6A). We noted that although CmpdA caused greater cleaved caspase-3 levels compared with Gefitinib treatment, the combination treatment showed a larger increase in cleaved caspase-3 expression. Since the cells grew slowly in the media, single treatment with Gefitinib or CmpdA did not show significant inhibition of cell proliferation, but the combination treatment showed greater inhibition than either single treatment (Supplementary Figure 6B). Our data indicated that synergy between IKK inhibition and Gefitinib will inhibit proliferation and survival in patient-derived HNSCC cells.

### CmpdA re-sensitised EGFR inhibitor-resistant cells to EGFR inhibitor

**Fig. 6:**
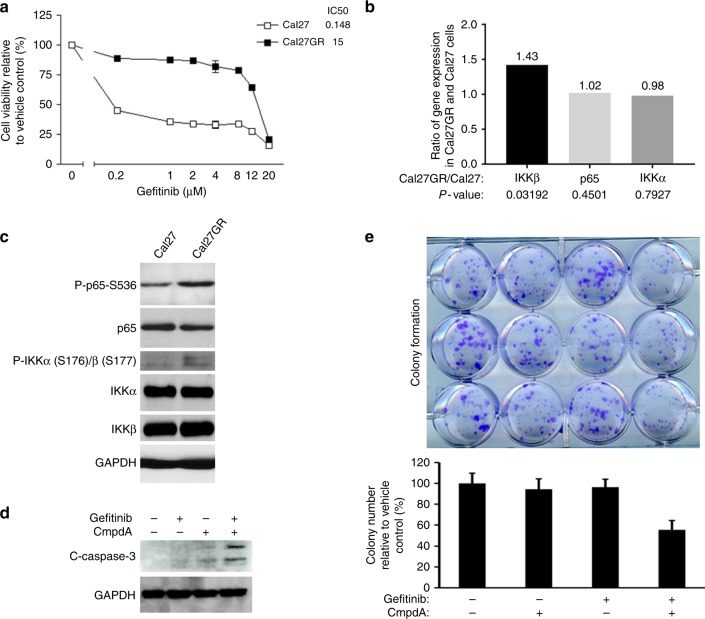
CmpdA re-sensitises Gefitinib-resistant HNSCC cells to Gefitinib. **a** The IC_50_ of Gefitinib was determined in Cal27 and Cal27GR cells. Cells were treated with cisplatin (0–100 μM) for 72 h at 37 °C. Error bars represent the standard error of means of three wells. **b** RNA was isolated from Cal27 and Cal27GR and a gene array experiment was performed. p65, IKKα and IKKβ expression in Cal27GP and Cal27 cells was compared. **c** IKKβ/NF-κB signalling is up-regulated in Cal27GR cells. Cal27 and Cal27GR were lysed and the phosphorylation and total levels of p65 and IKK were determined by Western blot. **d** Cal27GR cells were treated with DMSO, Gefitinib, CmpdA or a combination for 48 h and levels of cleaved caspase-3, were analysed by Western blot. **e** Cal27GR cells were treated with Gefitinib, CmpdA or a combination as indicated, and colony formation was observed 10 days after treatment

In order to study the role of IKK/NF-κB to regulate EGFR inhibitor resistance in head and neck cancers, we cultured Cal27 cells in media that initially contained 0.5 μM of Gefitinb, which was increased to 5 μM over a 6-month span. After 6 months of culture and selection, the cells were able to grow freely in media with 5 μM of Gefitinib. The MTS assay showed that the half-maximal inhibitory concentration (IC_50_) value was 15 μM for long-term Gefitinib-treated cells, which we named Cal27GR cells, whereas it was 0.15 μM for the parent Cal27 cells (Fig. 6a). Gene expression profiles showed that the mRNA levels of IKKβ in Cal27GR cells were much higher than that of Cal27 cells, whereas mRNA levels of both IKKα and NF-κB were equal (Fig. 6b). Western blot experiments demonstrated that the levels of both phospho-IKKβ and total IKKβ increased compared to that of parent cells. Consistent with increased IKKβ, p65 phosphorylation also increased (Fig. 6c). These data indicated that IKK, especially IKKβ, and NF-κB activities were up-regulated in Cal27GR cells. Gefitinib was not able to induce caspase-3 cleavage, and CmpdA induced low levels of caspase-3 cleavage; however, a combination induced greater caspase-3 cleavage (Fig. 6d). In line with the Western blot results, while CmpdA could inhibit colony formation, Gefitinib was unable to do so. The combination, however, led to significant inhibition of colony formation (Fig. 6e). Our data indicated that inhibition of IKK/NF-κB could overcome Gefitinib resistance in HNSCC cells.

## Discussion

**Fig. 7:**
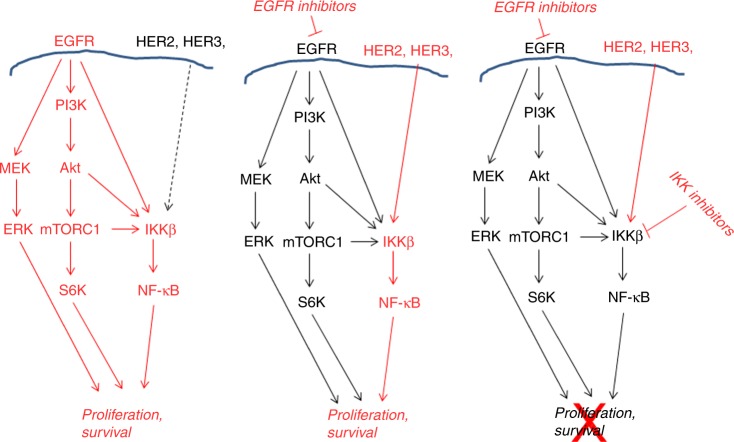
Model depicting pathways in response to Gefitinib and/or CmpdA treatment in HNSCC cells. Left and middle panels: EGFR is upstream of MEK/ERK, PI3K/Akt/mTOR and IKK/NF-κB pathways. EGFR inhibitors inhibited MEK/ERK and PI3K/Akt/mTOR but enhanced IKK/NF-κB pathways accompanied with increased HER2 and HER3 expression. Right panel: Inhibition of MEK/ERK, PI3K/Akt/mTOR and IKK/NF-κB pathways suppressed cell proliferation and survival by a combination of EGFR inhibitor with IKK inhibitor, CmpdA

PI3K/Akt/mTOR, MEK/ERK and IKK/NF-κB pathways are three crucial pathways downstream of EGFR signalling in HNSCC.^4,10^ We previously reported that PI3K/Akt/mTOR promoted IKK/NF-κB pathways through mTOR complex1 downstream of EGFR in HNSCC (25; Fig. 7, left panel). In this study, we examined the role of IKKβ/NF-κB on regulation of HNSCC cell sensitivity to EGFR kinase inhibitors, Gefitinib and Erlotinib. Interestingly, we found that inhibition of EGFR by Gefitinib or Erlotinib led to inactivation of PI3K/Akt/mTOR and ERK pathways, but up-regulated IKK/NF-κB signalling in multiple HNSCC cells (Fig. 7, middle panel). EGFR inhibition increased HER2 and HER3 levels, and also suppressed HER2 and HER3 phosphorylation. In addition, we determined that HER2 and HER3 were also involved in EGFR inhibitor induction of IKKβ/NF-κB (Fig. 7, middle panel). Our results suggested that IKKβ/NF-κB could play different roles in HNSCC proliferation and control of EGFR inhibitor resistance. Since the combination of EGFR and IKK inhibitors effectively blocked three crucial downstream pathways of EGFR, PI3K/Akt/mTOR, ERK and IKKβ/NF-κB, we concluded that the inhibitors synergised to induce apoptosis and suppress cell proliferation in HNSCC (Fig. 7, right panel). Our data implied that co-targeting EGFR and IKKβ/NF-κB signalling could be a potential novel therapy for head and neck squamous cell cancer for a subset of patients.

The mechanisms by which EGFR inhibitors induce IKK/NF-κB are complicated.^17–20^ It has been reported, both here and previously, that HER2 plays an important role in EGFR inhibitor resistance, and that EGFR inhibitors upregulate HER2 and HER3.^18,28,31,32^ Therefore, we treated HNSCC with the dual EGFR/HER2 inhibitor, Lapatinib. We found that Lapatinib was not able to block Gefitinib induction of IKK/NF-κB activation in HNSCC cell lines, including FaDU cells (Supplementary Figure 7). Interestingly, our data indicated that non-phosphorylated HER2 and HER3 play roles to regulate Gefitinib induction of IKK/NF-κB signalling. We found that while single depletion of HER2 or HER3 did not significantly impair Gefitinib induction of IKK/NF-κB, double depletion of HER2 and HER3 could do so. We also found an interaction between the HER2/3 heterodimer and IKKα/β complex (Fig. 4d). Our data implicate a crucial role for non-phosphorylated HER2 and HER3 in regulation of EGFR inhibitor resistance in HNSCC cells. It remains of utmost importance to determine the potential key mechanism by which the HER2/3 heterodimer promotes IKK activity upon EGFR inhibitor treatment.

Multiple groups have shown that Aurora-A kinase can contribute to EGFR inhibitor resistance through activation of NF-κB in several cancers.^18,33,34^ However, we found that treatment of FaDu cells with Alisertib, an Aurora-A inhibitor, could not abolish Gefitinib induction of NF-κB (Supplementary Figure 8). Likewise, we demonstrated that c-MET inhibitors could not effectively block NF-κB induction by Gefitinib in FaDu cells, although others reported that c-MET regulated EGFR inhibitor resistance through NF-κB in lung cancer (ref. ^35^, Supplementary Figure 9). Yet, other studies discovered that inhibition of EGFR signalling by Erlotinib induced NF-κB through NOX4 in HNSCC.^36,37^ It remains unclear whether NOX4 can mediate IKK activation in HNSCC. We found that HER2 and HER3 knockdown impaired Gefitinib-mediated IKK/NF-κB up-regulation, although the detailed mechanisms remain unclear. A study by Blakely et al.^38^ showed that Erlotinib both activated and regulated IKK/NF-κB through interaction with TRAF2. It would be interesting to determine whether or not HER2 and HER3 are also involved in this process. We are currently performing gene profile experiments to analyse any changes in gene expression between Cal27- and Gefitinib-resistant Cal27GR cells. In the future, we will examine whether those genes that are significantly up- or down-regulated in Cal27GR cells can also regulate IKK/NF-κB activation in response to EGFR inhibitor treatment.

An earlier important study by Pernas et al.^39^ reported that Gefitinib differentially affected the phosphorylation of Akt, ERK and STAT3 and suggested that Akt and STAT3 were crucial markers and therapeutic targets in HNSCC. This study showed that Gefitinib inhibited phosphorylation of NF-κB in UMSCC6 and UMSCC11A cells, but not in UMSCC9 and UMSCC11B cells after 4 h of treatment. Our data are consistent with theirs in regards to the UMSCC9 and UMSCC11B cells, but it should be noted that EGFR induction or inhibition of NF-κB could be cell-type-specific. Variations in dosage and time course of treatment may also display different responses in different cells and cancers. Furthermore, the effects of EGFR inhibitors on IKK/NF-κB also differ between basal and EGFR-induced IKK/NF-κB activation.

We noted that CmpdA, an IKKβ-specific kinase inhibitor, could not completely block NF-κB phosphorylation at Serine 536. It is possible that both IKKα and IKKβ contribute to NF-κB activation through phosphorylation of p65 at Serine 536; therefore, inhibition of IKKβ alone may lead to partial phosphorylation at this site. Furthermore, other kinases such as IKBKE (I-kappa-B kinase epsilon or IKK-epsilon) also regulate NF-κB phosphorylation and activation.^40,41^ Likewise, it may be possible that IKKα, IKKε and other NF-κB kinases play a role in activation of pro-survival pathways, which could abate the efficacy of CmpdA to inhibit cell proliferation and survival. Therefore, it is possible that a dual IKKα and IKKβ inhibitor, in combination with an IKBKE inhibitor, may completely block NF-κB at Serine 536 and improve EGFR inhibitor efficacy.

We also noted that CmpdA-induced IKKβ inhibition attenuated the ability of Gefitinib to inhibit Akt phosphorylation (Fig. 4a and Supplementary Figure 6A). We currently have no clear explanation for this result. In the future, we would like to determine whether IKKβ inhibition causes a compensatory activation of another signalling pathway to reactivate Akt independent of EGFR. It may also be important to examine whether the partial rebound of Akt phosphorylation caused by CmpdA attenuates the efficacy of a Gefitinib/CmpdA combination to inhibit cell proliferation and induction of apoptosis.

It should be mentioned that although the combination of Gefitinib and CmpdA was able to obviously suppress the tumour growth in vivo, the inhibitory efficacy for tumour volume in vivo was not as strong as in the in vitro clonogenic assay. There are several potential reasons for this occurrence. First, as stated in the Results section (Fig. 2), we observed significant necrosis in tumours in the control group that may have prevented us from obtaining more significant inhibitory effects in vivo. Additionally, the difference of the doses of Gefitinib in vitro and in vivo may also be a key factor. In our in vitro clonogenic assay, the concentrations of Gefitinib were 2 μM, whereas those in animal model were 15 mg/kg. We noted that, in many studies, the doses of Gefitinib for in vivo treatment were higher than 15 mg/kg, even reaching 100 mg/kg.^42,43^ Therefore, a dose higher than 15 mg/kg may achieve more significant inhibition of tumour growth.

Radiotherapy is another crucial tool for HNSCC treatment. It has been well documented that NF-κB confers sensitivity of HNSCC tumours to radiotherapy and has a direct association with patient prognosis.^44–46^ We would like to determine whether radiotherapy induces IKKβ kinase activity and whether CmpdA-induced inhibition of IKKβ improves the efficacy of radiotherapy in HNSCC.

This current study examined the effects of EGFR inhibitors on the phosphorylation of Akt, mTOR, ERK and IKK/NF-κB pathways. However, other pathways downstream of EGFR, such as STAT3 and PKC (protein kinase C) signalling, also play a role in HNSCC development and therapy resistance, and we will examine these factors in future studies.

## Electronic supplementary material

Supplementary data

## Availability of data and materials

The datasets used and analysed during the current study are available from the corresponding author on reasonable request.
