# Supplementary material for: Co-targeting EGFR and IKKβ/NF-κB signalling pathways in head and neck squamous cell carcinoma: a potential novel therapy for head and neck squamous cell cancer
Source: Br J Cancer. 2018 Dec 26;120(3):306–16. doi: 10.1038/s41416-018-0351-z (PMC6353914; doi:10.1038/s41416-018-0351-z)
Supplement: Supplementary file 1 — Supplementary data [file 41416_2018_351_MOESM1_ESM.docx]

**Supplementary figures with legends**

**Co-targeting EGFR and IKKβ/NF-κB signaling pathways in head and neck squamous cell carcinoma: a potential novel therapy for head and neck squamous cell cancer**

**Supplementary Figure 1**

**
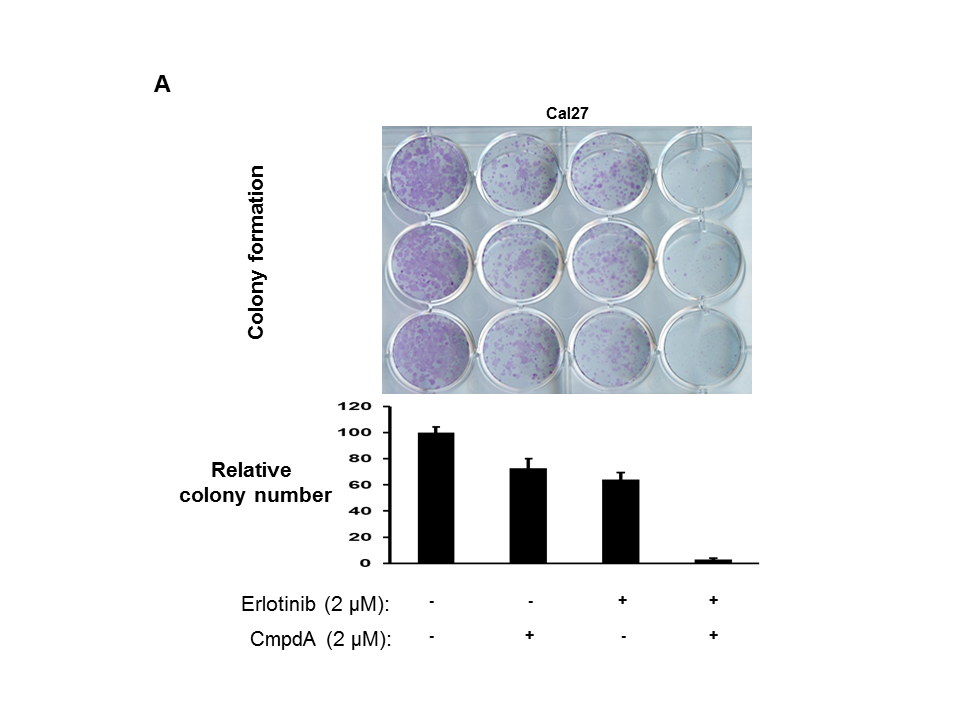
**

**
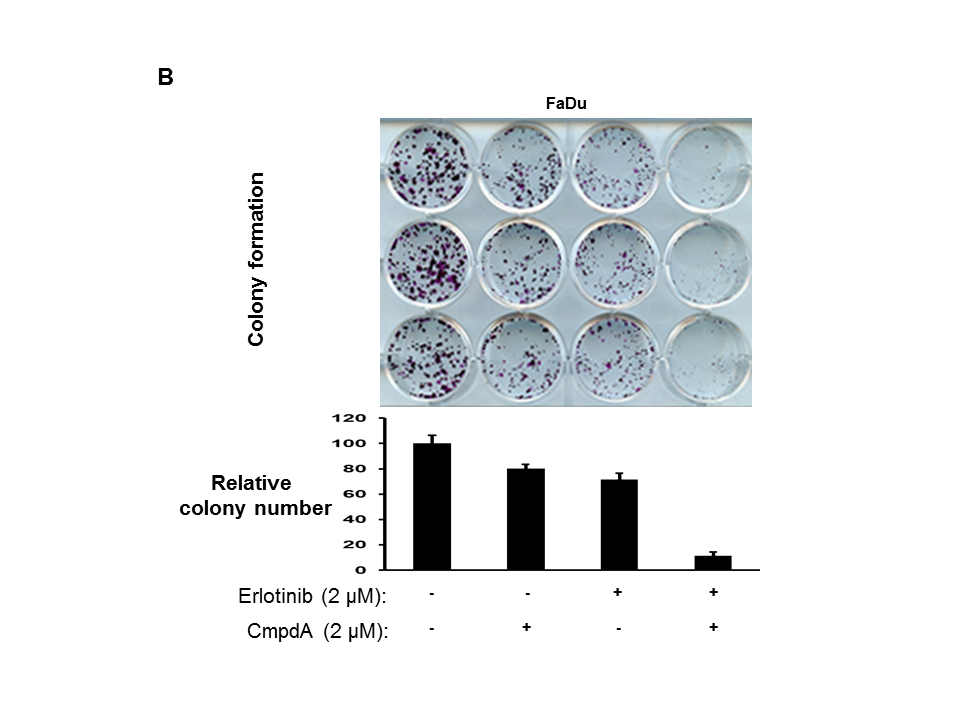
**

**Supplementary Figure 1, related to Figure 1**

**Inhibition of colony formation by combination of Erlotinib and CmpdA**

**A** and **B:** Synergistic inhibition of colony formation by a combination of Erlotinib and CmpdA. Cal27 (A) and FaDu (B) cells were treated with DMSO, Erlotinib, CmpdA, or a combination for 24 hours and colony formation was observed 10 days after treatment.

**Supplementary Figure 2**

**
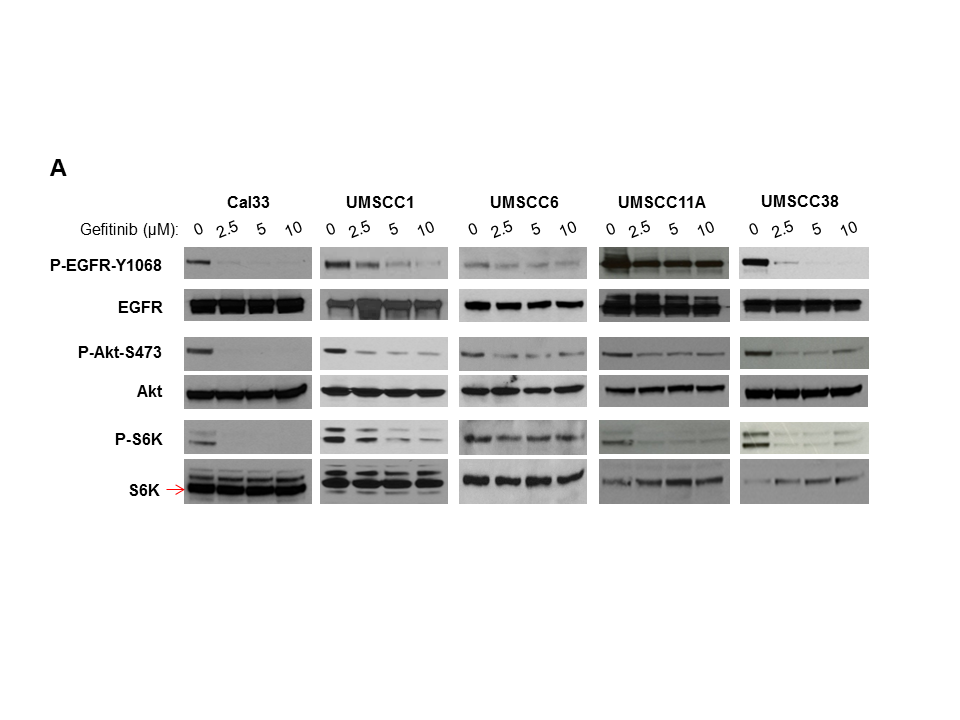
**

**
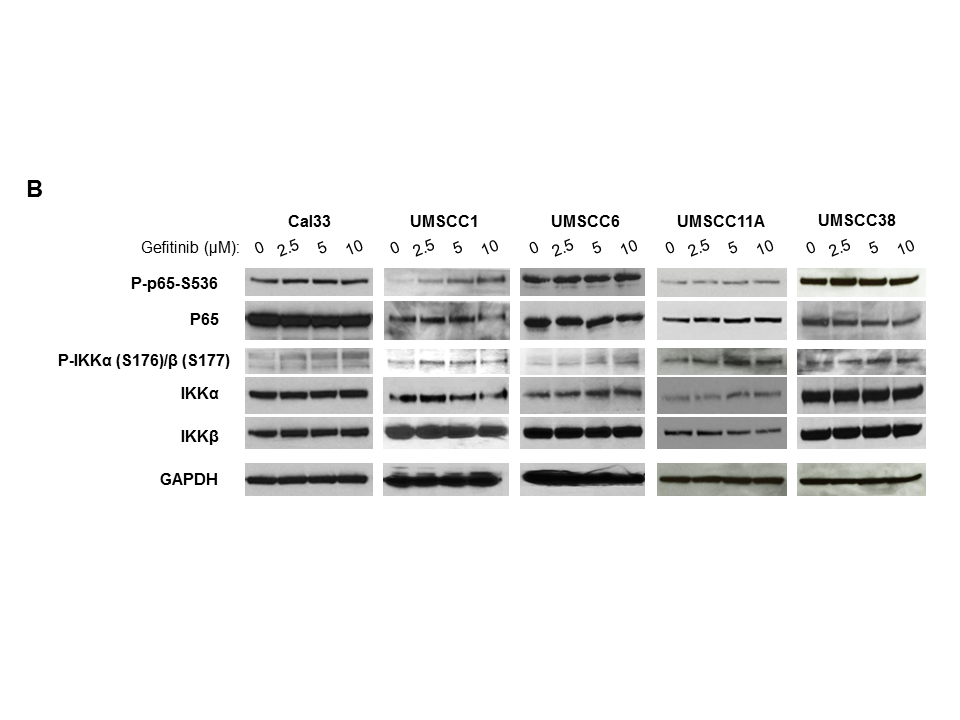
**

**Supplementary Figure 2, related to Figure 3**

**Gefitinib inhibits EGFR, Akt, mTOR, and ERK pathways but up-regulates IKKβ/NF-κB signaling in other HNSCC cell lines.**

**A**: Gefitinib inhibited EGFR, Akt, mTOR, and ERK phosphorylation. Multiple HNSCC cells were treated with vehicle control (DMSO) or different doses of Gefitinib (2, 5, or 10 μmol/L) for 24 hours, cells were lysed and phosphorylation and total protein levels of EGFR, Akt, S6K, and ERK were determined by Western blot analysis. **B:** Gefitinib enhanced phosphorylation of IKK and NF-κB. Cell lysates from **A** were analyzed by Western blot to test phosphorylation and total protein levels of IKKα/β and p65.

**Supplementary Figure 3**

**
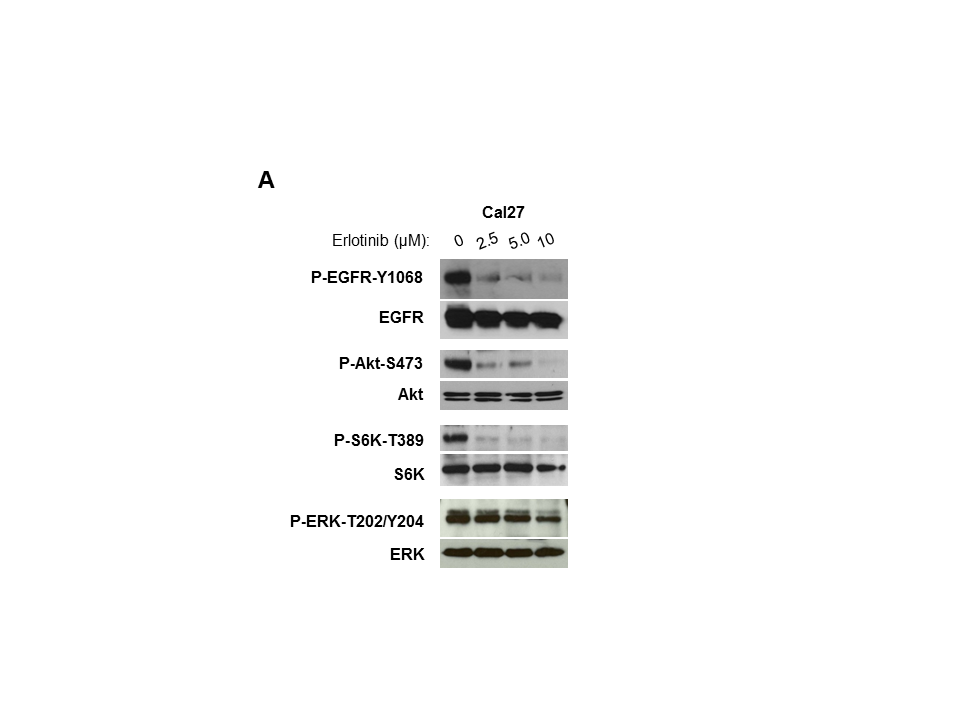
**

**
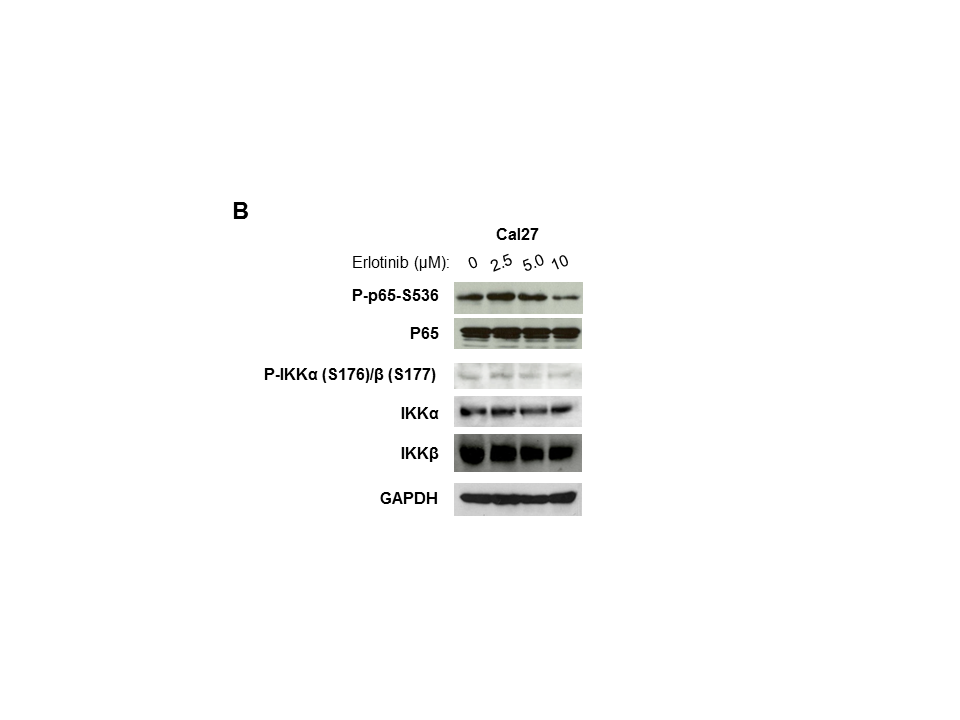
**

**Supplementary Figure 3, related to Figure 3**

**Erlotinib inhibits EGFR, Akt, mTOR, and ERK pathways, but up-regulates IKKβ/NF-κB signaling**

**A**: Erlotinib inhibits phosphorylation of EGFR, Akt, mTOR, and ERK pathways. Cal27 cells were treated with different doses of Erlotinib for 24 hours, cells were lysed and phosphorylation and total protein levels of EGFR, Akt, S6K, and ERK were determined by Western blot analysis. **B**: Upregulation of IKKβ and NF-κB phosphorylation by Erlotinib. Cell lysates from **A** were analyzed for phosphorylation and total IKKα/β and p65 protein levels.

**Supplementary Figure 4**

**
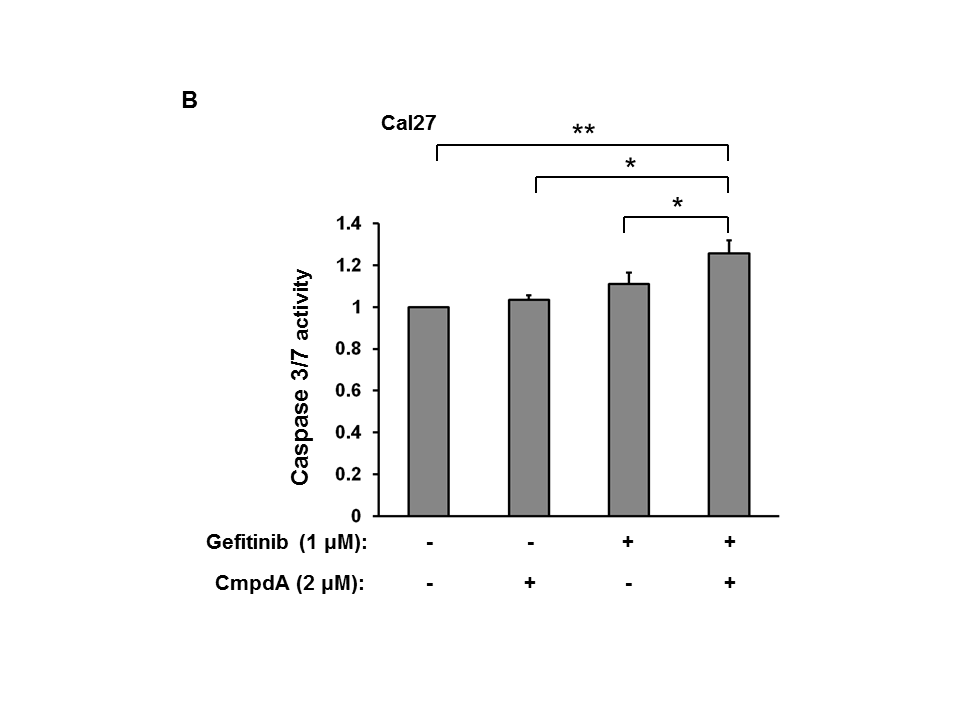
**

**
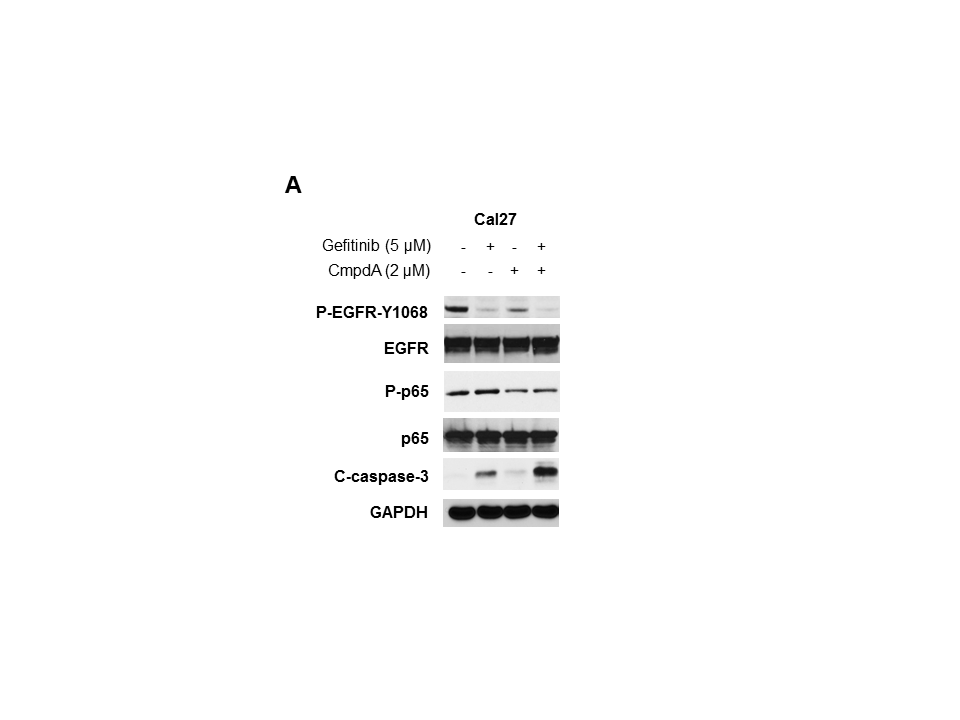
**

**
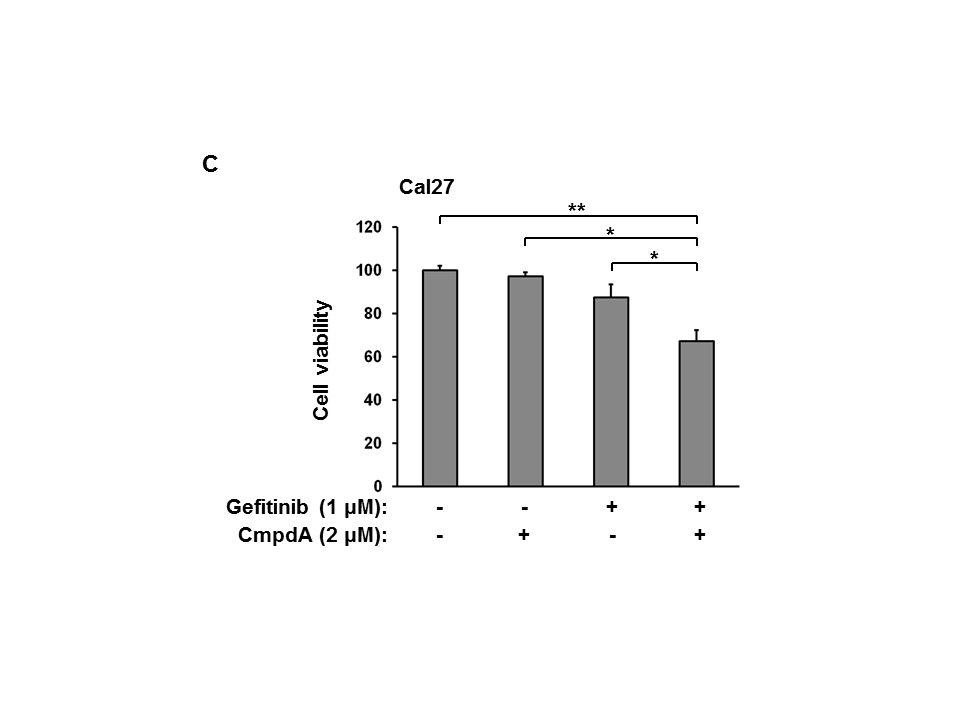
**

**Supplementary Figure 4, related to Figure 5**

**Inhibition of EGFR and NF-κB phosphorylation and induction of apoptosis by Gefitinib/CmpdA combination in Cal27 cells**

**A:** Inhibition of EGFR and NF-κB phosphorylation and induction of caspase-3 cleavage by a combination of Gefitinib and CmpdA. Cal27 cells were treated with DMSO, Gefitinib, CmpdA, or a combination for 48 hours and phosphorylation status of EGFR and p65, as well as cleaved caspase-3 levels, were analyzed by Western blot. **B:** Cells were treated as described above for 48 hours and caspase activity was measured. The experiments were performed in triplicate, and the results are representative of three independent experiments. **C**. Cells were seeded in 96-well plates for 24 hours and treated as described above for an additional 48 hours. Cell proliferation was determined by MTS assay (*p < 0.05; **p < 0.01).

**Supplementary Figure 5**

**
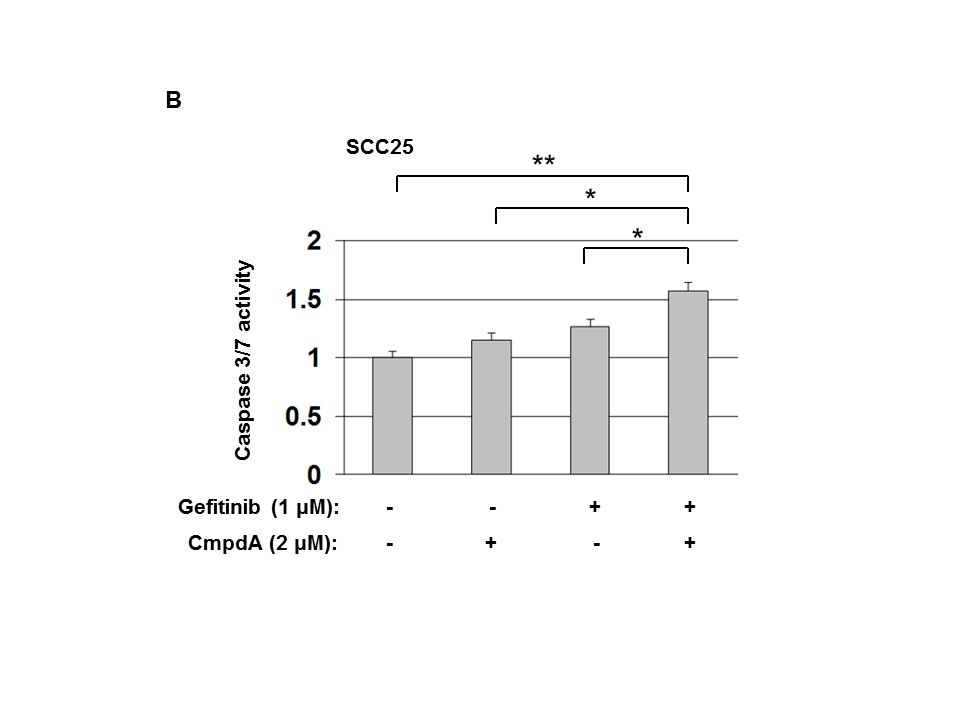

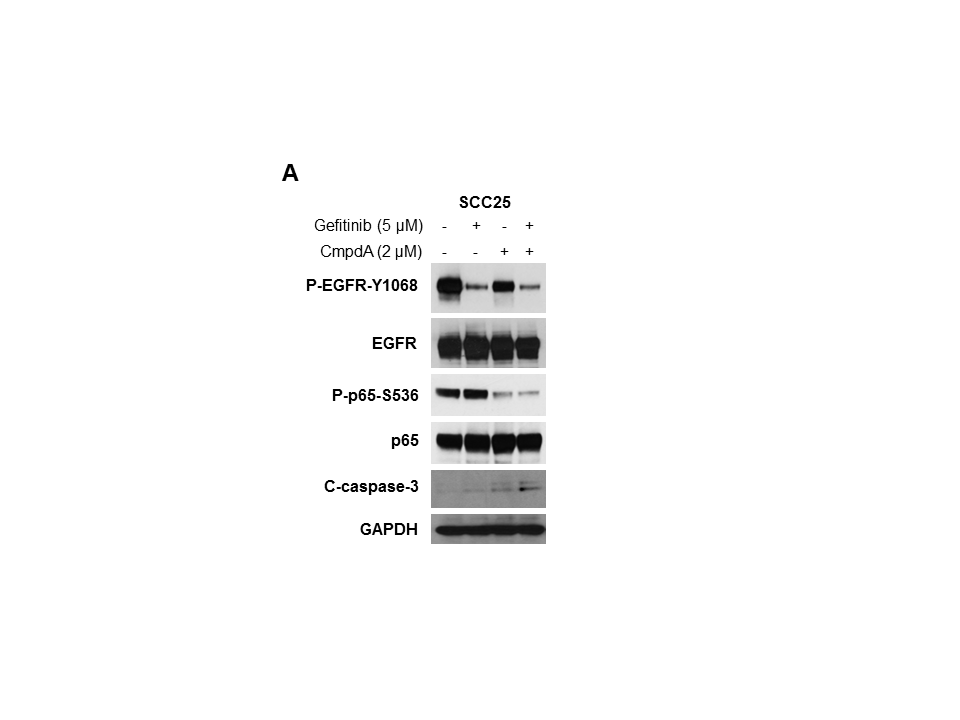
**

**
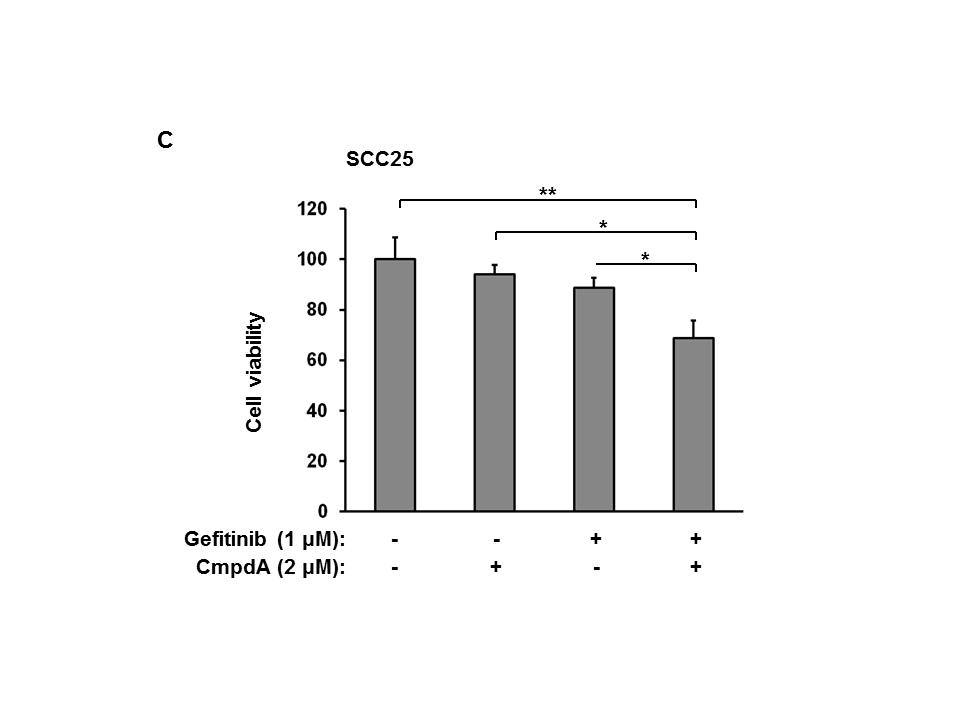
**

**Supplementary Figure 5, related to Figure 5**

**Inhibition of EGFR and NF-κB phosphorylation and induction of apoptosis by Gefitinib and CmpdA combination in SCC25 cells**

**A:** SCC25 cells were treated with DMSO, Gefitinib, CmpdA, or a combination for 48 hours and phosphorylation status of EGFR and p65, as well as cleaved caspase-3 levels, were analyzed by Western blot. **B:** Cells were treated with DMSO, Gefitinib, CmpdA, or a combination for 48 hours and caspase activity was measured. The experiments were performed in triplicate, and the results are representative of three independent experiments. **C**: Cells were treated with DMSO, Gefitinib, CmpdA, or a combination for 48 hours and cell proliferation was determined by MTS assay (*p < 0.05; **p < 0.01).

**Supplementary Figure 6**

**
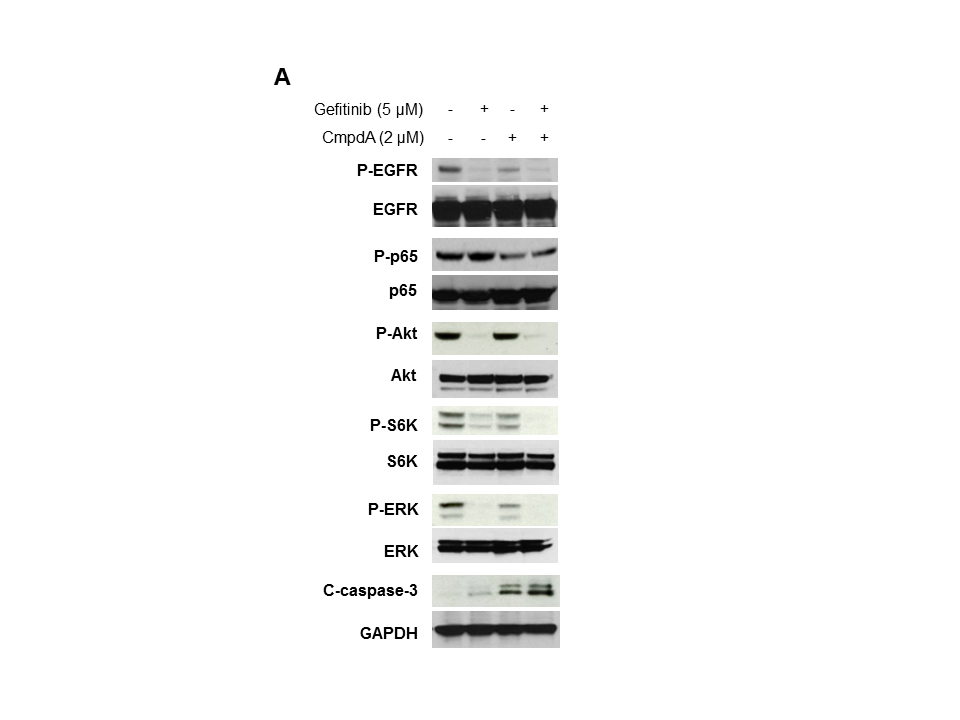
**

**
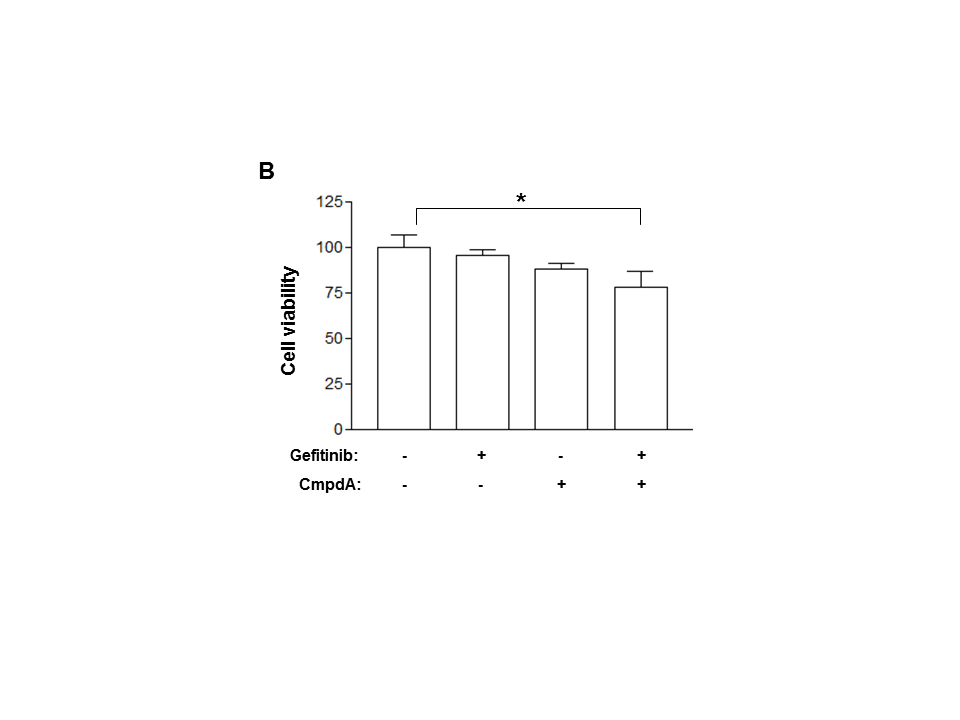
**

**Supplementary Figure 6, related to Figure 5**

**Combination of Gefitinib and CmpdA inhibits patient-derived HNSCC cell proliferation**

**A:** Cells were cultured in media with 10% FBS and growth factors for 24 hours and then treated with DMSO, Gefitinib, CmpdA, or a combination for 48 hours. Phosphorylation and total protein levels of p65, Akt, S6K, and ERK, as well as levels of cleaved caspase-3, were analyzed by Western blot. **B:** Cells were seeded in 96-well plates for 24 hours and treated as described above for an additional 72 hours. Cell proliferation was determined by MTS assay (*p < 0.05).

**Supplementary Figure 7**

**
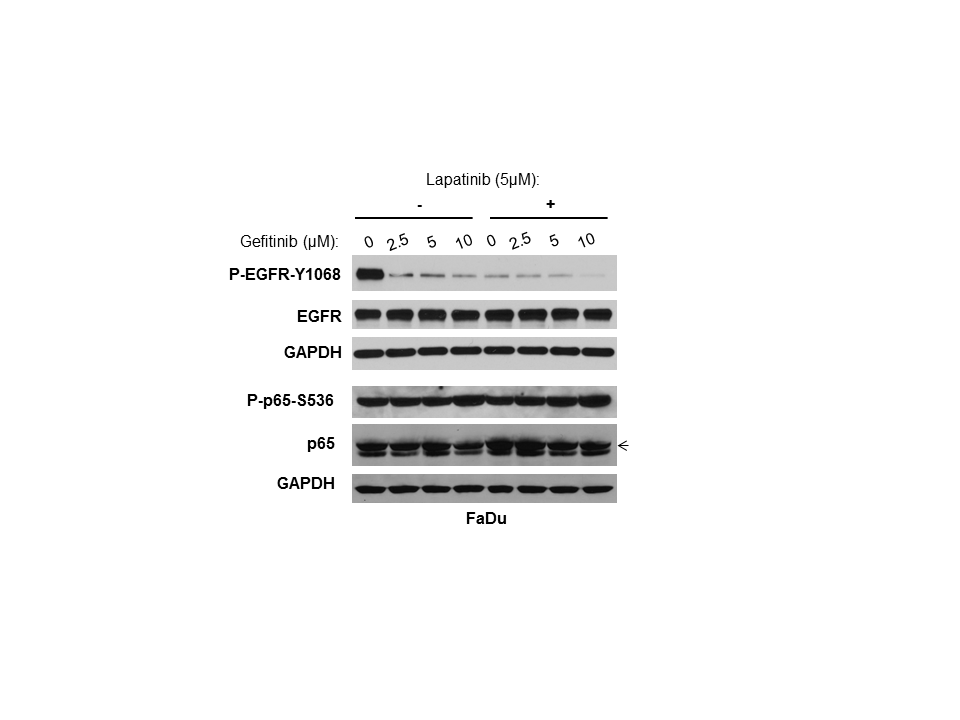
**

**Supplementary Figure 7**

**Lapatinib is not able to inhibit Gefitinib-induction of NF-κB phosphorylation**

Cells were treated with increasing doses of Gefitinib or in combination with Lapatinib for 24 hours. Cells were lysed and phosphorylation and total protein levels of EGFR and p65 were determined by Western blot analysis.

**Supplementary Figure 8**

**
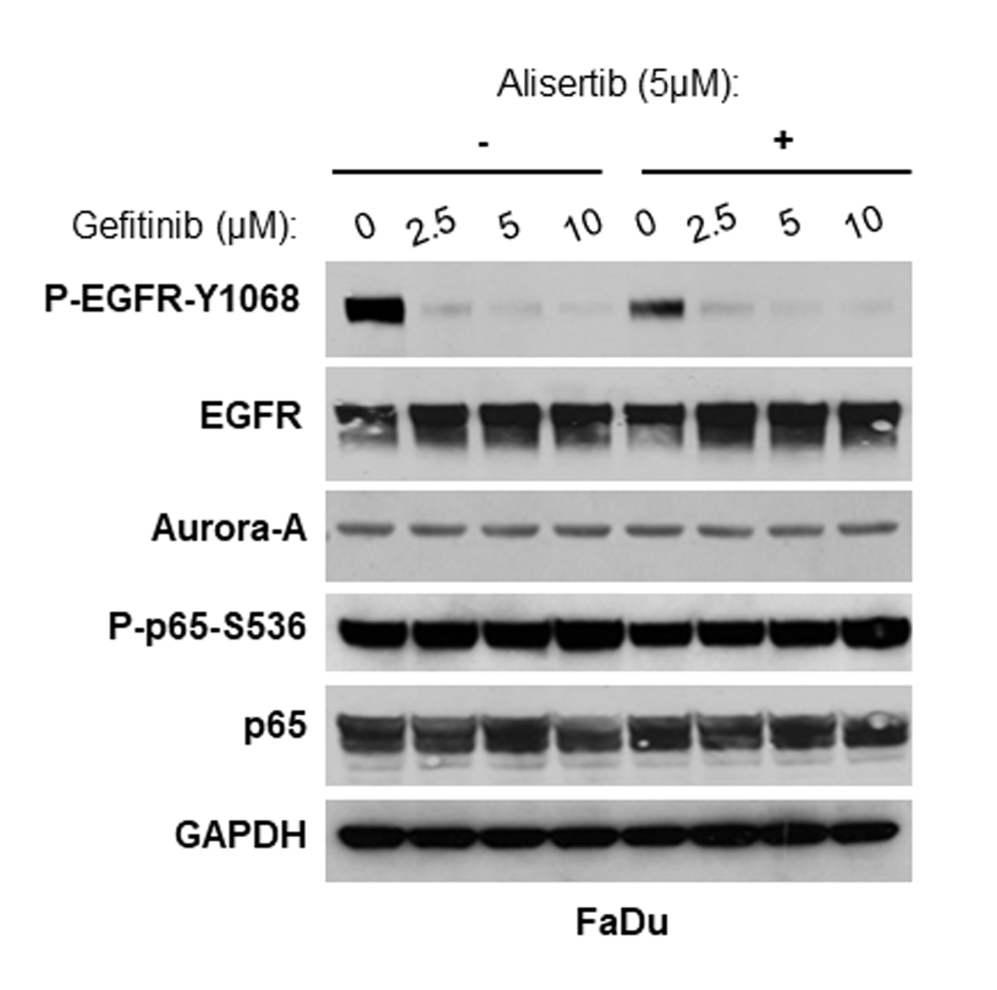
**

**Supplementary Figure 8**

**Aurora-A inhibitor, Alisertib, is not able to inhibit Gefitinib-induction of NF-κB phosphorylation**

Cells were treated with increasing doses of Gefitinib or in combination with Alisertib for 24 hours. Cells were lysed and phosphorylation and total protein levels of EGFR, Aurora-A, and p65 were determined by Western blot analysis.

**Supplementary Figure 9**

**
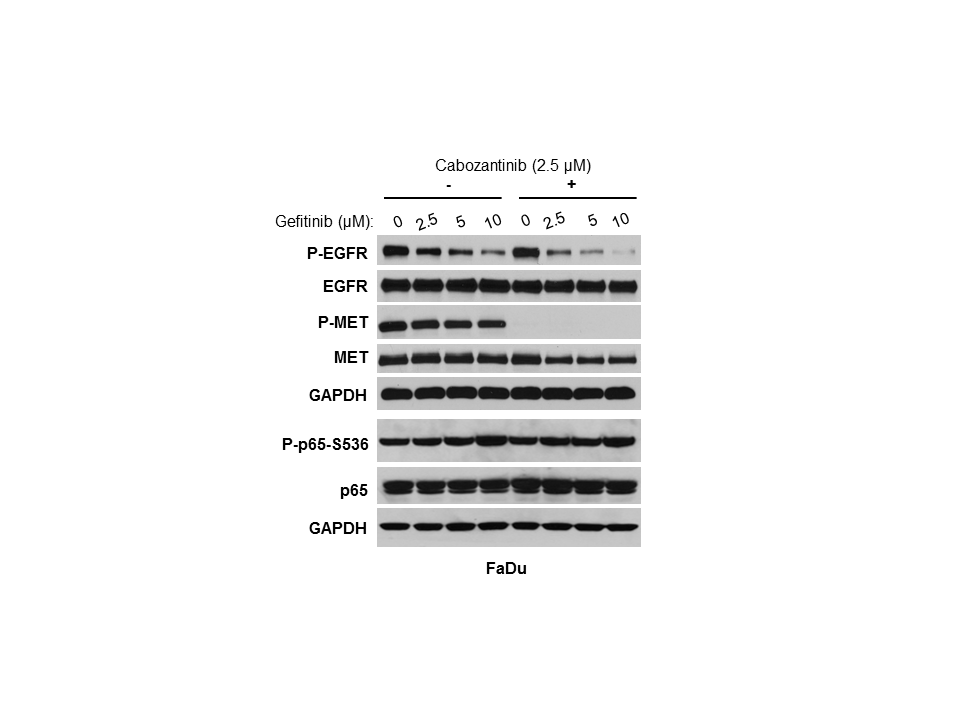
**

**Supplementary Figure 9**

**C-MET inhibitor, Cabozantinib, is not able to inhibit Gefitinib-induction of NF-κB phosphorylation**

Cells were treated with increasing doses of Gefitinib or in combination with Cabozantinib for 24 hours. Cells were lysed and phosphorylation and total protein levels of EGFR, c-MET, and p65 were determined by Western blot analysis.
